# Survival and nutritional status of children with severe acute malnutrition, six months post-discharge from outpatient treatment in Jigawa state, Nigeria

**DOI:** 10.1371/journal.pone.0196971

**Published:** 2018-06-20

**Authors:** Collins John, Udochukwu Diala, Ruth Adah, Luret Lar, Esther Awazzi Envuladu, Idris Adedeji, Kazeem Lasisi, Oluseyi Olusunde, Femi James, Halima Abdu

**Affiliations:** 1 Department of Paediatrics, Jos University Teaching Hospital, Jos, Nigeria; 2 Department of Community Medicine, Jos University Teaching Hospital, Jos, Nigeria; 3 Department of Paediatrics, Abubakar Tafawa Balewa University Teaching Hospital Bauchi, Bauchi, Nigeria; 4 Department of Mathematical Sciences, Abubakar Tafewa Balewa University Bauchi, Bauchi, Nigeria; 5 National Primary Health Care Development Agency, North Central Zonal Office, Abuja, Nigeria; 6 Federal Ministry of Health, Maternal, Newborn and Child Health Unit, Abuja, Nigeria; TNO, NETHERLANDS

## Abstract

**Background:**

The Outpatient Therapeutic Program (OTP) for treatment brings the management of Severe Acute Malnutrition (SAM) closer to the community. Many lives have been saved through this approach, but little data exists on the outcome of the children after discharge from such programmes. This study was aimed to determine the survival and nutritional status of children at six months after discharge from OTP for SAM.

**Methodology:**

This was a prospective study of children with SAM admitted into 10 OTPs in two local government areas of Jigawa state from June 2016 to July 2016.

Home visits at six months after discharge enabled the collection of data on survival and nutritional status.

The primary outcome measures were survival and nutritional status (Mid upper arm circumference and weight-for-height z-score).

**Result:**

Of 494 children with SAM, 410 were discharged and 379 were followed up. Of these, 354, (93.4%) were found alive while 25 (6.6%) died. Among the survivors 333 (94.1%) had MUAC ≥12.5cm and 64 (18.1%) had WHZ<-3.

Mortality rates were higher 10 (8.4%) among the 6-11months old. Most deaths 16 (64%) occurred within the first 3months post-discharge. Those who died were significantly more stunted, p = 0.016 and had a smaller head circumference, p = 0.005 on entry to OTP programme.

There was improvement from admission to six months follow up in the number of children with complete immunization (27.4% to 35.6%), and a decrease in the number of unimmunized children (34.8% vs 20.6%) at follow-up.

**Conclusion:**

The study demonstrates good post discharge survival rate and improved nutritional status for SAM patients managed in OTPs. There were, however considerable post discharge mortality, especially in the first three months and lower immunization uptake post discharge. A follow-up programme will improve these indices further.

## Introduction

The Outpatient Therapeutic Program (OTP) brings the management of Severe Acute Malnutrition (SAM) closer to the community by making services available at decentralized treatment points within the Primary Health Care (PHC) settings [1]. This is achieved through community mobilization by Community Volunteers (CVs),) and the use of ready-to-use therapeutic foods. The OTP is a part of the wider Community Management of Acute Malnutrition (CMAM). Treatment for children with complications is provided by inpatient management in stabilization centres as the need arises [2].

Admission and discharge into and from OTP depends on set guidelines drawn from the World Health Organization guidelines. This includes the use of Mid-Upper Arm Circumference (MUAC) of <11.5cm at admission and >12.5cm at discharge [3].

While many lives have been saved through CMAM, little data exists about the outcome of the children after discharge from such centres [4] as no follow up plan is included in the CMAM programme, except for those with specific problems identified during inpatient treatment of cases [4–7].

In Nigeria, there is little or no report on the outcome of children treated in CMAM clinics. The long-time survival outcome is also unknown among these children and reports [4] indicate poor outcome on a long term basis. In a bid to add to the existing body of knowledge on the outcome of SAM patients especially post-discharge, this study examined the survival, nutritional and immunisation status of children with SAM who had been treated as outpatient, six months post-discharge.

### Ethical approval

Approval for the study was obtained from the Jigawa State Primary Health Care development agency. Written and signed informed consent was obtained from caregivers of the children before recruitment.

## Methodology

The study was conducted in two randomly selected Local Government Areas (LGAs) in Jigawa State, documented as one with a high prevalence of malnutrition [8]. Of 12 LGAs implementing CMAM, 3 LGAs; Birnin Kudu, Gwiwa and Gurin are supported by *Action Against Hunger* (ACF) to implement the CMAM programme [7]. Two of these three were randomly selected and in the selected local government areas, five CMAM clinics were randomly selected using random number tables from each LGA.

### Study design

This study was a prospective study between June 2016 and April 2017. Children aged 6–59 months were recruited from CMAM clinics at presentation and followed-up at home 6 months after discharge.

#### Admission and discharge criteria

Admission into the CMAM programme was based on a MUAC <11.5cm, no oedema or with minimal oedema and no apparent medical conditions.

Discharge criteria were a MUAC ≥12.5cm on two consecutive visits, one week apart in line with the *Action Against Hunger* (ACF) guideline [9].

The clinics are managed by four to five community health extension workers (CHEW), with some training in CMAM, and 25–75 community volunteers (CVs) involved with community mobilization of SAM patients for treatment.

#### Sample size determination

A minimum sample size was determined using the formula below. [10]

$$n=\frac{z^{2}(pq)}{d^{2}}$$

Where n = minimum sample size

z = standard deviation score at 95% confidence interval (1.96)

p = prevalence of wasting in Jigawa State [11]

q = complement probability (1-p)

d = Absolute precision (error tolerance = 0.05)

The calculated sample size using the prevalence of wasting in Jigawa State, 17.7% [11] was 226.3.

SPHERE{14} minimum default rate of 10% was added, 22.6 = 249.

This was multiplied by two to give 498 as total subjects to be recruited. Each CMAM centre was allocated 50 subjects in both local governments.

#### Study population

The admission criteria for entry into the CMAM programme met by the children. Of these children, study subjects were randomly selected each day using a random number table until the calculated sample size was reached. Each week, in each site, eight to ten subjects were selected for recruitment. (The clinics runs CMAM programme once a week) All subjects recruited received care as provided in the clinic. This included group counselling on hygiene and feeding; antibiotics; immunization and age-appropriate doses of ready-to use therapeutic food and weekly clinic visit until discharge. Indivdually, a review of child immunization status and futher counselling was done and subjects were offered appropriate immunization. The research team did not interfere in admission or discharge of subjects from the clinics. Recruitment of study subjects was done over a period of one month from June 2016 to July 2016. Recruitment was discontinued after the first discharge from the clinic was recorded.

### Data collection

Standard anthropometric measurements were taken from all subjects at the recruitment, and at follow-up after discharge from the clinic. MUAC was measured using a color-coded MUAC tape on the left arm. Length was measured using an infantometer mat while weight was measured using a Seca 877 mother-child digital scale. Head circumference (HC) was measured using a non-stretchable tape. All measurements were entered into the android device. To ensure quality of data, range check and double entry of measurements were done.

Derived anthropometric measurements Weight for Height (WHZ), Height for Age (HAZ) and Weight for Age z scores (WAZ) were obtained for each child.

Responses to survey questions at the time of entry and follow up were recorded using the Census and Survey Processing (CSPro) software system, developed by the U.S. Census Bureau and ICF Macro.

At entry to the OTP information on bio-data, marital and educational status of parents, occupation, and Infant and Young Child Feeding (IYCF) practices was recorded and dietary diversity using a 3-day recall was obtained. Immunization and medical history were obtained. At six months post-discharge, information on IYCF, illness and immunization during the period of study was also captured. Important events that occurred between the periods of discharge and follow-up were also recorded.

### Data analysis

The data collected was analysed using STATA 14.0MP. Frequency tables, was used to show results with the level of significance set at p <0.05. Derived anthropometric indices were calculated using the STATA zscore06 macro, reflecting the WHO multi-centre growth reference standard (WHO-MGRS) [12].

## Results

A total of 494 children were recruited of which 410 (83%) were discharged from the clinic and of these 379 (93.4%) were found during follow up home visit at 6months after discharge. The subjects not followed up had either relocated, gave untraceable addresses or were nomadic herdsmen and hence could not be tracked at the time of the visit.

### Flow chart of subjects

**Fig 1:**
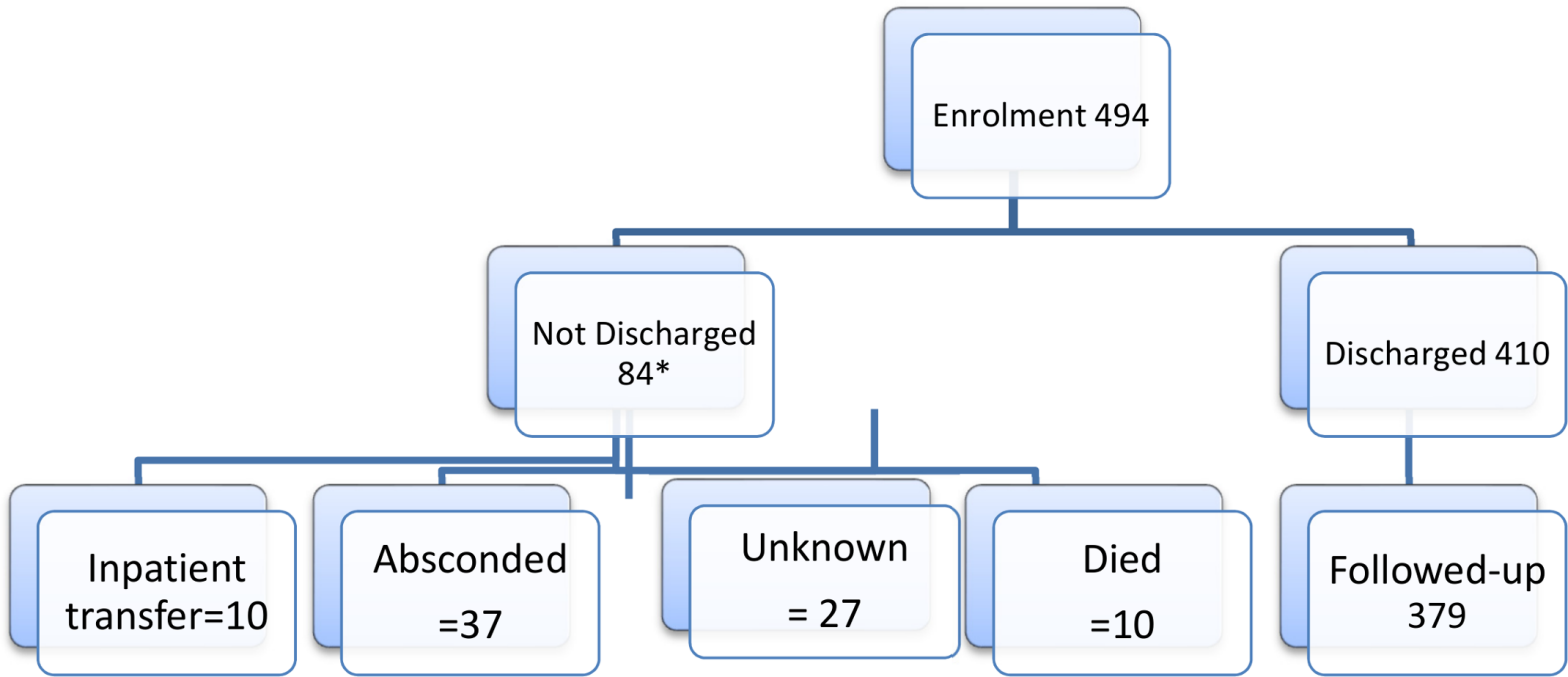
Flow chart of subjects.

The flow chart of subjects from enrolment to follow up is shown in Fig 1 (Flow Chart of Subjects)

### General characteristics

Among the 379 subjects followed-up, males were 182 (48%) and females 197 (52%). Of this 354 (93.4%) were alive at the home visit 6months after discharge. The others, 25 (6.6%) died.

Among mothers of subjects, 171 (57.2%) were between 20-29years of age and most, 230 (60.7%) had an Arabic education while 52 (13.7%) had primary education. Ages of mothers were reported in 299 of mothers. This is shown in Table 1 below.

**Table 1:** Maternal age, parental education status and place of child birth.

Variables	Frequency	Percent
**Maternal age groups**	n = 299	
<19	21	7.0
20–29	171	57.2
30–39	85	28.4
40+	22	7.4
**Educational status of mother**	n = 379	
None	82	22.6
Arabic	230	60.7
Primary	52	13.7
Others	15	3.0

Mean Maternal age 26.7±6.7

The mean duration of clinic stay and mortality rate is shown in Table 2 below. The mean duration of clinic attendance was 51.2±11.2 days. Mortality was higher (10%) in subjects who stayed less than 6weeks and those who stayed above 9weeks

**Table 2:** Duration of OTP treatment and survival.

Duration	Alive		Died		Total		% Mortality (Died/Total)
	n	%	n	%	n	%	
**4-5weeks**	**54**	**15.3**	**6**	**24.0**	**60**	**15.8**	**10.0**
**6-7weeks**	**117**	**33.0**	**5**	**20.0**	**122**	**32.2**	**4.1**
**8weeks**	**120**	**33.9**	**7**	**28.0**	**127**	**33.5**	**5.5**
**9weeks+**	**63**	**17.8**	**7**	**28.0**	**70**	**18.5**	**10.0**
**Total**	**354**	**100.0**	**25**	**100.0**	**379**	**100.0**	**6.6**

Mean duration of clinic stay 51.2±11.2days (28-84days)

The table below (Table 3) shows the distribution of mortalities by age group at recruitment. Mortality was highest in the youngest age group at recruitment, 6-11months, 10 (8.4%).

**Table 3:** Outcome by age at recruitment.

Age at recruitment (months)	Number at follow up	Number dead at Follow up	% Mortality
6–11	119	10	8.4
12–23	169	8	4.7
24–35	89	7	7.9
36–47	2	0	0
Overall	379	25	6.6

### Place and time of death

The deaths were recorded more at home as shown in **Table 4**. In all, 64% of the deaths took place within three months after discharge. Four died one month after, six in second and third month, four each in the fourth and fifth month and two were in the sixth month.

**Table 4:** Place and timing of death.

Place	Frequency	Percentage
Home	19	76.0
Road to hospital	2	8.0.
Health Facility	4	16.0
Time		
1-3month	16	64,0
4-6month	9	36.0
Total	25	100.0

From a univariate analysis, significant differences between predictors of mortalities are reported in Table 5 below. Children who died were significantly more stunted and had a smaller mean Head Circumference (HC) at admission. MUAC was similar in each group

**Table 5:** Recruitment anthropometric characteristics and survival status.

Variables	Dead (CI)	Alive (CI)	p value
Height	65.3 9.3	69.1 6.6	0.008
HAZ	-4.6±2.4	-3.5±1.8	0.005
WHZ	-2.66±2.4	-3.4±1.9	0.057
WAZ	-4.4±1.3	-4.3±0.9	0.8
MUAC	10.78±0.8	10.8±0.5	0.8
Mean HC	42.7±2.4	44.2±2.2	0.002
HC 6-11months	41.4±2.0	42.5±1.7	0.04*
HC 12-23months	42.6±1.7	44.6±1.9	0.005*
HC 24-35months	44.8±2.4	45.4±2.0	0.44*
Oedema-Yes	3 (11.5%)	11 (3.1%)	0.13

*sub-categories of mean HC

The nutritional status of the children at entry, discharge and follow up is shown in Table 6 below. Significant changes occurred in nutritional status from the time of admission to the time of the home visit, 6months after discharge.

**Table 6:** Nutritional status at recruitment, discharge and follow-up.

Nutritional status	Recruitment		Discharge		Follow-up		p value
**MUAC (cm)**	**n**	**%**	**n**	**%**	**n**	**%**	
≥12.5	0	0.0	351	92.6	333	93.1	<0.0001
11.5–12.4	0	0.0	20	5.3	18	5.1	
<11.5	379	100.0	8	2.1	3	0.8	
Total	379	100.0	379	100.0	354	100.0	
***WHZ***	***n***	***%***	***n***	***%***	***n***	***%***	***p value***
<-3	261	68.9	96	25.3	64	18.1	<0.0001
<-2	61	16.1	74	19.3	61	17.2	
>-2	57	15.0	210	55.4	229	64.7	
Total	379	100.0	379	100.0	354	100.0	

Among the subjects, there were significant changes in immunization status between admission and six-months follow up. The proportion of children who had completed immunization increased from 27.4% to 35.6%. The number of un-immunized children dropped from 34.8% to 20.6%. This is shown in Table 7 below.

**Table 7:** Immunization status by phase.

	Recruitment		Post Discharge	
Immunization status*	n	%	n	%
**Up-to-date/complete**	**104**	**27.4**	**126**	**35.6**
**Incomplete**	**143**	**37.7**	**155**	**43.8**
**Un-immunized**	**132**	**34.8**	**73**	**20.6**
**Total**	**379**	**100.0**	**354**	**100.0**

*Oral and card review

## Discussion

Our study of follow up at 6 months after discharge from OTP for SAM showed that 6.6% had died, 18.1% had remaining wasting and 43.6% were incompletely immunised. At present, the protocols do not include follow up visits after discharge from OTP for SAM. Thus this study seeks to add to existing knowledge and influence CMAM programme policy to include post discharge follow up, at least 6months after exit from SAM therapy in the community.

The study highlights the post discharge outcome of patients with SAM. This is the first of such report from Nigeria that provides data on the survival and nutritional status of children admitted and treated for SAM at 6 months after discharge.

The mortality rate in this study among successfully discharged patients from the CMAM programme of 6.6% is higher than the 3.7% reported by Bahwere P *et al* [13] in a meta-analysis of a community based programme. However, it is lower than the Sphere standard for emergency and humanitarian situations at discharge, put at 10% [14]. Our mortality rates are much lower than reported by Kerac M *et al* even though their cohort high percentage of children with untreated HIV [4]. The study by Kerac M *et al* reported a mortality rate of 17% in children without HIV infection [4]. Although the HIV status of our subjects are unknown, Jigawa state has a very low HIV prevalence of 1.5%, lower than that of most states in Nigeria and the national average (3.2%) [15].

In a study of follow up after inpatient treatment for SAM in Bangladesh, a mortality rate of 8.7% at 3 months after discharge was reported by Chisti MJ *et al*. [16]. Though not similar cohorts, we noted that most deaths in our study were within the first three months post discharge similar to the findings in Bangladesh [16]. This is also similar to the report, by Wiens et al in a study, on pediatric post discharge mortality in resource settings among a cohort that included malnourished children [7]. They reported that most post-discharge deaths occurred early during the post-discharge period. The difference however is while our subjects were outpatients, the Wiens et al review was among inpatients [7].

The nutritional status at recruitment between the deceased subjects and survivors differed significantly, especially the HAZ and head circumference. Children who died in our study were significantly more stunted and had smaller head circumference, especially in the age groups 6–11 and 12–23 months at recruitment. Similarly Chisti MJ *et al* also found a higher mortality in severely malnourished children [16].

We observed that the high rates of improved MUAC levels (>12.5cms) at discharge (92.6%) were maintained at follow-up (93.1%). We also noted that there was improvement in the proportion of children with WHZ < 3 at follow up (18.1%) compared with the proportion at discharge (25.3%).

Although WHZ was not the admission criteria, we however, noted that the rates are not concordant with MUAC <11.5cm prevalence rates. This is accordance with a previous report by John C *et al* [17].

There was also an improvement in the uptake of immunization as the prevalence of children with completed/upto-date immunization was higher in post-discharge than at recruitment. The frequency of un-immunized children dropped significantly from 34.8% to 20.6%. The change in immunization status can be attributed to attendance at the clinic, which provided opportunities for immunization gaps to be covered. This was demonstrated in a study by Kalathia *et al* [18]. Though the drop in prevalence of un-immunized subjects is good, there are significant numbers of missed opportunities for immunization. The finding suggests that interest in immunization wanes as subjects exit the CMAM programme, this ought not to be as it is envisaged that contact with a health clinic such as the CMAM centres will cause an increased uptake and interest in immunization. This appears not so hence efforts need to be geared towards community/home based vaccine administration. This method of has been shown in Ghana to result in improved uptake of immunization [19]. The missed opportunities are connected with ease of access as shown by Jani JV *et al* in rural Mozambique among children less than two years [20]. Opportunities for community delivered immunization eg combined with CMAM programmes will be an added advantage to outcome and childhood development in such areas [19].

### Strengths and limitations of the study

This study draws strength from the prospective nature of the design. It is the first of such studies among malnourished children in Nigeria. The sample size was reasonably large and the study has added to the understanding of the pattern of mortality among successfully discharged SAM patients. It also draws attention to differences between mortality outcome by anthropometric criteria of MUAC and WHZ.

This study however was conducted shortly before the peak of the rainy season and seasonal variations cannot be extrapolated as this may play a significant role in outlook of malnutrition.

## Conclusion and recommendation

The study demonstrates good post-discharge outcome with a survival rate of 93.4%, improved nutritional status among subjects. Published mortality rates after discharge following OTP for SAM vary considerably. While ours are higher than those described by Bahwere *et al* [13], they are lower than those published by Kerac *et al* [4].

Higher mortality rate, especially among the younger age groups; those staying less than six weeks; and those staying longer than eight weeks require a review of the discharge criteria. The extremely difficult social, cultural, political and economic situation in northern Nigeria makes the provision and uptake of health and nutrition care to be very challenging.

We conclude that follow up visits after discharge need to be included and its impact evaluated.

## Supporting information

S1 Data collection tool Study questionnaire. (DOCX)

S1 Dataset Recruitment data file. (DTA)

S2 Dataset Discharge data file. (DTA)

S3 Dataset Follow-up data file. (DTA)

S4 Dataset Merged All phases data file. (DTA)

S5 Dataset Edited follow-up data file. (DTA)
