# Supplementary material for: Survival and nutritional status of children with severe acute malnutrition, six months post-discharge from outpatient treatment in Jigawa state, Nigeria
Source: PLoS One. 2018 Jun 20;13(6):e0196971. doi: 10.1371/journal.pone.0196971 (PMC6010258; doi:10.1371/journal.pone.0196971)
Supplement: S1 Data collection tool — (DOCX) [file pone.0196971.s001.docx]

# EVALUATING THE OUTCOMES AND IMPACT OF CARE OF CHILDREN MANAGED FOR SEVERE ACUTE MALNUTRITION IN NORTHERN NIGERIA

1. ENROLMENT

Date: No: [baseline] [

## GENERAL DATA

Name of LGA and PHC/OTP

1. LGA-BIRNIN KUDU
2. KANGIREI PHC
3. YELWAN DAMAI PHC
4. SAMAMIYA PHC
5. UNGWAN YA PHC
6. WURNO PHC
7. LGA-GWIWA
8. FIRJI PHC
9. YOLA PHC
10. DABI PHC
11. GWIWA MPHC
12. KORAYAL PHC

## BIODATA

Name of Community child coming from.....................................?

Catchment communities/ ward/villages ……………………….....

Full Description of home address of child:…………………………………………… ………………………………………………………………………………………………………………………………………………………………………………………………………………………………………

- Name of primary care giver................................................
- Relationship to child…………………drop box—mother, father, brother, sister, aunt, uncle, grandparent, neighbour, others specify……………………….…..
- Phone Number of caregiver ……......................................
- Name of Secondary caregiver care giver..................................
- Relationship to child
- Number of secondary care giver

## SOCIODEMOGRAPHIC DATA

1. Name of Child (3 names)…………………………………………….
2. Age (months)……………………………………………...
3. Date of Birth ( optional)
4. Gender a) Male b) Female
5. State of Origin…………………………………………….
6. Ethnicity...a. hausa---- b, fulani----c. Yoruba......d. igbo.......e. indigenious tribe f. others specify.....………………………………………………………
7. Religion………christian, islam, traditional, others specify……………………………………

### FAMILY DATA

1. Family size/ No of people living in the household…………………………………………..
2. Type of household: a) Nuclear b) Extended
3. Marital status of parents or caregiver: a) Married b)Single c) Separated
   1. d) Divorced e) Widowed
4. mothers order of marriage
5. Type of marriage: a) monogamous b. Polygamous
6. Type of Family: a) Nuclear b) Extended
7. How many children does the mother have? .............................................................................
8. Number of children aged 5 years and under in the household(...................................
9. Number of sleeping/ bed rooms in house occupied by family..................................
10. Birth order of the child: ……………………………………………………………………...
11. Place of delivery: a) Health facility b) TBA c) home
    1. d) Others (specify)………………………………….
12. What is mother’s age? ………………………………………………
13. age of mother at first marriage.....
14. Age at first pregnancy...............................
15. What is father’s age?.....................................................................
16. What is the highest completed level of education attained by the mother? a) None b. Arabic/Primary education c) junior secondary d. Secondary education e) Higher Institution below University f) University education
17. What is the highest level of education attained by the father

a) None b. Arabic/ Primary education c) junior secondary (d). Secondary education e) Higher Institution below University f) University education

1. What is mother’s occupation? None....farming......trader......artisan(specify)... junior public servant, senior public servant..........others specify
2. What is father’s occupation? None....farming......trader......artisan(specify)... junior public servant, senior public servant..........others specify
3. What is mother’s monthly income?
   1. <6000 [ ] 6000- <12, 000 [ ] 12 000-<18,0000 [ ] 18000- 36,000[ ] >36000-48000[ ] > 48 000[ ]
4. What is father’s monthly income?
   1. <6000 [ ] 6000- <12, 000 [ ] 12 000-<18,0000 [ ] 18000- 36,000[ ] >36000-48000[ ] > 48 000[ ]
5. Is the child living with the parents? a) Yes b) No
6. If not living with parents, who does the child live with? Relations [ ] Friends [ ] Adopted [ ] Institutionalised [ ] ..............................................

## DIETARY HISTORY

1. Did the mother ever breast feed this child? a) Yes b) No
2. What did the child receive in the first 6 months of life? a) Breast milk alone b) Breast milk and water c) Breast milk and infant formula d) Infant formula only e) Others (specify) ……………………………………………….
3. For how long was this child given breast milk alone? ...................................................
4. How soon after delivery was breastfeeding started for the child? a) 0-30 minutes b) 31-60 minutes c) >60 minutes< 24hrs d) >24 hours
5. In the first three days of life, did the child receive the milk that flowed from the mother’s breast (colostrum)? a) Yes b) No
6. Have you stopped breast feeding the child? a) Yes b) No
7. At what age did you introduce semi solid/solid food to the child? …………………………..
8. Have you ever received any education on infant and young child feeding?

a) Yes b) No

1. Who decided that this child should come to cmam clinic......... dropbox... mother, father, paternal grandmother, paternal grandfather, paternal grand aunt......maternal,,,,,...... co-wife......... drop box
2. 3 days food recall................................
   1. Carbohydrates a. Bread [ ] b. Rice [ ] c. Indomie [ ] d. Sweet potatoes[ ] e. Yam [ ] f. Cassava [ ]g. Plantain [ ] h. Cocoyam [ ] I. Garri [ ] j. Plantain [ ]
   2. Proteins a. Beef [ ]b. Pork [ ] c. Goat [ ] d. Chicken[ ] e. Duck [ ] f. Fresh fish[ ] g. Dry fish [ ] h. Shellfish[ ] i. Sea food[ ] J. Eggs[ ] k. Liver [ ] l. Kidney[ ] m. Heart[ ] n. Beans product[ ] o. Cheese[ ] p. Yoghurt [ ]q. Others[ ]
   3. Oil and fats a. Vegetable oil [ ] b. Butter[ ] c. Red palm oil [ ] d. Others...............
   4. Fruits and vegetables a. Mango[ ] b. Pawpaw[ ] c. Banana [ ]d. Orange[ ] e. Apple[ ]. f.garden egg [ ] g. Carrot [ ] h. Pear[ ] i Water melon [ ]. Others.
   5. Snacks a. Chocolates[ ] b. Sweets[ ] c. Pastries[ ] d. Biscuits [ ]e. Pastries[ ].

## MEDICALHISTORY

1. Immunization given. ......a. dropbox....... 0..... 6 weeks.......10 weeks..........14weeks.........9months.........
2. Has the child been ill at any time last one month yes ..... no .....................
3. If yes for how long............................(days)
4. What were the symptoms of the illness ?( tick as appropriate)fever...... cough........ fast breathing..........vomiting......... watery stool..........bloody stool .....refusal to feed ........ convulsion.....weakness........body swelling ......yellowness of eyes.......skin rash ......

## PHYSICAL EXAMINATION

1. Oedema: a) Present b) Absent
2. Has the child been given RUTF / plumpy /pro nut today/ first day of admission Y/N

## ANTHROPOMETRYOFCHILD

1. Weight 1 ……………….kg Weight 2……….……….kg Mean………..….kg
2. Height/Length 1………..cm Height/Length 2………..cm Mean…………..cm
3. MUAC 1………………..mm MUAC 2………..……..mm Mean…………..mm
4. WAZ Z-score……………….
5. WHZ Z-score………….…….
6. HAZ Z Score………..
7. PCV……………
8. RDT……………
9. Urinalysis…………..

# B . DISCHARGE

1. Date…….
2. How many weeks spent in cmam clinic in total
3. age as of discharge
4. Address…… a )same b. changed, if changed …..where?.........................................
5. Has the child been ill many days of last one month yes ..... no .....................
6. If yes for how long............................(days)
7. What were the symptoms of the illness? (tick as appropriate) fever...... cough........ Fast breathing.......... vomiting......... watery stool.......... bloody stool..... Refusal to feed........ Convulsion..... Weakness........ Body swelling ...... yellowness of eyes....... skin rash......

## PHYSICAL EXAMINATION

1. Oedema: a) Present b) Absent

## ANTHROPOMETRYOFCHILD

1. Weight 1 ……………….kg Weight 2……….……….kg Mean………..….kg
2. Height/Length 1………..cm Height/Length 2………..cm Mean…………..cm
3. MUAC 1………………..mm MUAC 2………..……..mm Mean…………..mm
4. WAZ Z-score……………….
5. WHZ Z-score………….…….
6. HAZ Z-score………..…… these by the data

# C. 6MTH FOLLOW UP

1. Date as of interview…….
2. alive----, dead-----

if alive……. To continue with interview, IF NOT go to section on death

1. age as of follow up (mths)……….
2. Has the child been ill since discharge from cmam clinic yes ..... no .....................
3. If yes how many times did he have any of these
4. What were the symptoms of the illness ?( tick as appropriate)fever...... cough........ fast breathing..........vomiting......... watery stool..........bloody stool .....refusal to feed ........ bleeding..........convulsion.....weakness........body swelling ......yellowness of eyes.......skin rash ...... others specify........................................
5. Since discharge how many times has the child been admitted into hospital for illnesses.........
6. 3 Day dietary recall
   1. Carbohydrates a. Bread [ ] b. Rice [ ] c. Indomie [ ] d. Sweet potatoes[ ] e. Yam [ ] f. Cassava [ ]g. Plantain [ ] h. Cocoyam [ ] I. Garri [ ] j. Plantain [ ]
   2. Proteins a. Beef [ ]b. Pork [ ] c. Goat [ ] d. Chicken[ ] e. Duck [ ] f. Fresh fish[ ] g. Dry fish [ ] h. Shellfish[ ] i. Sea food[ ] J. Eggs[ ] k. Liver [ ] l. Kidney[ ] m. Heart[ ] n. Beans product[ ] o. Cheese[ ] p. Yoghurt [ ]q. Others[ ]
   3. Oil and fats a. Vegetable oil [ ] b. Butter[ ] c. Red palm oil [ ] d. Others...............
   4. Fruits and vegetables a. Mango[ ] b. Pawpaw[ ] c. Banana [ ]d. Orange[ ] e. Apple[ ]. f.garden egg [ ] g. Carrot [ ] h. Pear[ ] i Water melon [ ]. Others.
   5. Snacks a. Chocolates[ ] b. Sweets[ ] c. Pastries[ ] d. Biscuits [ ]e. Pastries[ ].

## PHYSICAL EXAMINATION

1. Oedema: a) Present b) Absent

## D

## ANTHROPOMETRYOFCHILD

1. Weight 1 ……………….kg Weight 2……….……….kg Mean………..….kg
2. Height/Length 1………..cm Height/Length 2………..cm Mean…………..cm
3. MUAC 1………………..mm MUAC 2………..……..mm Mean…………..mm
4. WAZ Z-score……………….
5. WHZ Z-score………….…….
6. HAZ Z-score………..…… these by the data

## C2. DEAD CHILD DURING FOLLOW UP

IF CHILD IS DEAD ……. ,………………………………………………..

1. did the respondent live with child at time of death…yes…no

2. . When did the child die ( months after discharge)…dropbox—1,2,3,4,5,6

3. Where did the child die…….home, health facility,en route to health facility, others specify

4. Healthcare sought before death………………….none, health facility, chemist, traditional healers, prayer houses , others specify…….multiple options

5. What season did the child die…………………………….rainy, dry
